# Supplementary material for: Social anxiety and academic performance during COVID-19 in schoolchildren
Source: PLoS One. 2023 Jan 12;18(1):e0280194. doi: 10.1371/journal.pone.0280194 (PMC9836262; doi:10.1371/journal.pone.0280194)
Supplement: Attachment — Submitted filename: Changes Letter.docx [file pone.0280194.s001.docx]

Dear Reviewers,

Thanks for your suggestions in the improvement of the manuscript. The recommendations of the three reviewers have been followed. According to that, below you can see a list of the corrections that have been made:

**Journal Requirements:**

The participants gave their written informed consent. The document from line 123 to 128 talks about it.

The performance of the funders in the study has been included in the cover letter.

**Reviewer 1**

- To be more precise in the valuable insights of this study it was added in lines 39-42 the surplus of the study as well as in the last paragraph of the introduction.
- In lines 43- 67 it was stated the current state of art in mask usage and their impact.
- Considering the COVID effect it was added the period in which the study was carried out in the introduction. See lines 67-69.
- In addition, in the design section it was clarified the period in which the study was carried out to highlight that it was after lockdown and in a compulsory mask usage.

**Reviewer 2**

-The title was shortened according to the suggestion. Thanks so much.

- Lines 12-14. The phrase was shortened to be more precise.

- Line 71. It was used the word “in comparison to” to do not be redundant.

- In the introduction. It was added the surplus of the study in the lines 39-40 as well as in the last paragraph of the introduction.

- Lines 111-112. It was cited that the classification taken was from the following study:

22. Caballo VE, Arias B, Salazar IC, Calderero M, Irurtia MJ, Ollendick TH. Un nueva medida de autoinforme para evaluar la ansiedad/fobia social en niños: el "Cuestionario de ansiedad social para niños"(CASO-N24). Behavioral Psychology/Psicología Conductual. 2012;20(3):485-504.

- The table 1 was completed according to the suggestions made.

- The manuscript was revised to correct typos and to abide by the Vancouver style.

**Reviewer 3**

-The reference to "sports variables" in Table 2 has been removed.

-A quotation has been added in the introduction that show the relationship between social anxiety and academic performance.

-In the first paragraph of the discussion (218-223) has been discussed why schoolchildren with high social anxiety worsened their overall grades in Physical Education.

-The names of citation 36: Miranda, Margoth, Pantoja and Valdivieso have been removed.
